# Supplementary material for: Time-series transcriptome analysis identified differentially expressed genes in broiler chicken infected with mixed Eimeria species
Source: Front Genet. 2022 Aug 8;13:886781. doi: 10.3389/fgene.2022.886781 (PMC9393255; doi:10.3389/fgene.2022.886781)
Supplement: Supplementary file 2 [file DataSheet1.ZIP › 7dpi_KEGG.Gsea.1625115961950/KEGG_OXIDATIVE_PHOSPHORYLATION.html]

Details for gene set KEGG\_OXIDATIVE\_PHOSPHORYLATION[GSEA]

|  || Dataset | TMM\_7dpi\_gct\_format\_7dpi\_gct\_format.Class\_4dpi.cls #PC\_versus\_NC.Class\_4dpi.cls #PC\_versus\_NC\_repos |
| Phenotype | Class\_4dpi.cls#PC\_versus\_NC\_repos |
| Upregulated in class | 1 |
| GeneSet | KEGG\_OXIDATIVE\_PHOSPHORYLATION |
| Enrichment Score (ES) | 0.79219234 |
| Normalized Enrichment Score (NES) | 3.899516 |
| Nominal p-value | 0.0 |
| FDR q-value | 0.0 |
| FWER p-Value | 0.0 |
Table: GSEA Results Summary

  

Fig 1: Enrichment plot: KEGG\_OXIDATIVE\_PHOSPHORYLATION      
 Profile of the Running ES Score & Positions of GeneSet Members on the Rank Ordered List

  

| SYMBOL | TITLE | RANK IN GENE LIST | RANK METRIC SCORE | RUNNING ES | CORE ENRICHMENT || 1 | ATP6V1C2 | na | 4 | 2.128 | 0.0424 | Yes |
| 2 | ATP6V0A4 | na | 19 | 1.686 | 0.0752 | Yes |
| 3 | ATP6AP1 | na | 21 | 1.657 | 0.1084 | Yes |
| 4 | ATP6V0D2 | na | 24 | 1.636 | 0.1411 | Yes |
| 5 | ATP6V0C | na | 30 | 1.603 | 0.1729 | Yes |
| 6 | ATP6V1D | na | 46 | 1.458 | 0.2010 | Yes |
| 7 | ATP12A | na | 48 | 1.456 | 0.2302 | Yes |
| 8 | ATP6V1E1 | na | 61 | 1.395 | 0.2572 | Yes |
| 9 | ATP6V0E1 | na | 72 | 1.354 | 0.2836 | Yes |
| 10 | ATP6V1H | na | 80 | 1.313 | 0.3094 | Yes |
| 11 | ATP6V0B | na | 100 | 1.252 | 0.3330 | Yes |
| 12 | ATP6V1G3 | na | 102 | 1.247 | 0.3580 | Yes |
| 13 | COX5A | na | 300 | 0.942 | 0.3604 | Yes |
| 14 | NDUFA2 | na | 303 | 0.935 | 0.3790 | Yes |
| 15 | NDUFB6 | na | 307 | 0.933 | 0.3975 | Yes |
| 16 | NDUFS5 | na | 308 | 0.932 | 0.4162 | Yes |
| 17 | NDUFB7 | na | 323 | 0.915 | 0.4335 | Yes |
| 18 | NDUFB2 | na | 348 | 0.885 | 0.4492 | Yes |
| 19 | NDUFAB1 | na | 356 | 0.876 | 0.4663 | Yes |
| 20 | NDUFA7 | na | 371 | 0.857 | 0.4823 | Yes |
| 21 | NDUFC2 | na | 394 | 0.834 | 0.4972 | Yes |
| 22 | UQCR10 | na | 444 | 0.785 | 0.5089 | Yes |
| 23 | COX6A1 | na | 480 | 0.760 | 0.5212 | Yes |
| 24 | UQCR11 | na | 485 | 0.754 | 0.5361 | Yes |
| 25 | UQCRB | na | 487 | 0.753 | 0.5511 | Yes |
| 26 | NDUFA11 | na | 497 | 0.746 | 0.5654 | Yes |
| 27 | COX7A2 | na | 499 | 0.743 | 0.5802 | Yes |
| 28 | NDUFS3 | na | 623 | 0.675 | 0.5835 | Yes |
| 29 | NDUFB3 | na | 656 | 0.657 | 0.5940 | Yes |
| 30 | NDUFS8 | na | 661 | 0.653 | 0.6068 | Yes |
| 31 | NDUFS6 | na | 686 | 0.642 | 0.6177 | Yes |
| 32 | SDHD | na | 708 | 0.632 | 0.6286 | Yes |
| 33 | NDUFA1 | na | 742 | 0.616 | 0.6382 | Yes |
| 34 | UQCRHL | na | 788 | 0.594 | 0.6464 | Yes |
| 35 | NDUFA5 | na | 794 | 0.593 | 0.6579 | Yes |
| 36 | SDHB | na | 840 | 0.575 | 0.6657 | Yes |
| 37 | NDUFB5 | na | 902 | 0.554 | 0.6717 | Yes |
| 38 | COX6C | na | 930 | 0.546 | 0.6804 | Yes |
| 39 | NDUFV3 | na | 988 | 0.527 | 0.6862 | Yes |
| 40 | NDUFB4 | na | 1015 | 0.517 | 0.6944 | Yes |
| 41 | COX7B | na | 1055 | 0.501 | 0.7012 | Yes |
| 42 | NDUFA6 | na | 1079 | 0.492 | 0.7092 | Yes |
| 43 | NDUFA8 | na | 1080 | 0.492 | 0.7191 | Yes |
| 44 | UQCRQ | na | 1103 | 0.484 | 0.7270 | Yes |
| 45 | NDUFS2 | na | 1124 | 0.477 | 0.7349 | Yes |
| 46 | COX4I1 | na | 1125 | 0.476 | 0.7445 | Yes |
| 47 | SDHC | na | 1135 | 0.473 | 0.7532 | Yes |
| 48 | NDUFB10 | na | 1215 | 0.450 | 0.7557 | Yes |
| 49 | UQCRFS1 | na | 1263 | 0.437 | 0.7605 | Yes |
| 50 | ATP5PB | na | 1267 | 0.436 | 0.7690 | Yes |
| 51 | NDUFV1 | na | 1307 | 0.428 | 0.7743 | Yes |
| 52 | NDUFB8 | na | 1330 | 0.423 | 0.7810 | Yes |
| 53 | NDUFS7 | na | 1371 | 0.412 | 0.7859 | Yes |
| 54 | NDUFV2 | na | 1395 | 0.408 | 0.7922 | Yes |
| 55 | UQCRC2 | na | 1700 | 0.332 | 0.7734 | No |
| 56 | COX7A2L | na | 1761 | 0.316 | 0.7747 | No |
| 57 | COX11 | na | 1823 | 0.302 | 0.7757 | No |
| 58 | NDUFB9 | na | 1842 | 0.299 | 0.7802 | No |
| 59 | COX15 | na | 1927 | 0.282 | 0.7788 | No |
| 60 | NDUFB1 | na | 1995 | 0.272 | 0.7786 | No |
| 61 | UQCRC1 | na | 2326 | 0.217 | 0.7554 | No |
| 62 | ATP6V1G1 | na | 2444 | 0.199 | 0.7495 | No |
| 63 | LHPP | na | 2509 | 0.189 | 0.7480 | No |
| 64 | NDUFA4 | na | 2726 | 0.160 | 0.7331 | No |
| 65 | NDUFA9 | na | 2879 | 0.141 | 0.7232 | No |
| 66 | COX10 | na | 3408 | 0.073 | 0.6804 | No |
| 67 | NDUFA10 | na | 3412 | 0.073 | 0.6816 | No |
| 68 | SDHA | na | 3488 | 0.064 | 0.6766 | No |
| 69 | COX17 | na | 3545 | 0.059 | 0.6731 | No |
| 70 | NDUFS1 | na | 3763 | 0.033 | 0.6556 | No |
| 71 | PPA2 | na | 3783 | 0.031 | 0.6546 | No |
| 72 | TCIRG1 | na | 3866 | 0.020 | 0.6481 | No |
| 73 | ATP6V1C1 | na | 4112 | -0.007 | 0.6277 | No |
| 74 | NDUFS4 | na | 4402 | -0.040 | 0.6043 | No |
| 75 | ATP6V0A1 | na | 4522 | -0.052 | 0.5954 | No |
| 76 | PPA1 | na | 4532 | -0.054 | 0.5957 | No |
| 77 | COX7C | na | 4881 | -0.090 | 0.5684 | No |
| 78 | ATP6V0D1 | na | 5522 | -0.151 | 0.5178 | No |
| 79 | ATP6V1A | na | 5917 | -0.192 | 0.4886 | No |
| 80 | ATP6V1B2 | na | 5973 | -0.199 | 0.4880 | No |
| 81 | ATP6V0E2 | na | 6836 | -0.290 | 0.4216 | No |
| 82 | ATP6V0A2 | na | 9318 | -0.602 | 0.2257 | No |
Table: GSEA details [plain text format]

  

Fig 2: KEGG\_OXIDATIVE\_PHOSPHORYLATION      
 Blue-Pink O' Gram in the Space of the Analyzed GeneSet

  

Fig 3: KEGG\_OXIDATIVE\_PHOSPHORYLATION: Random ES distribution      
 Gene set null distribution of ES for **KEGG\_OXIDATIVE\_PHOSPHORYLATION**
